# Anaesthesia Management of a Case with Hereditary Angioedema for Whom Tracheal Dilatation was Planned

**DOI:** 10.4274/TJAR.2025.241584

**Published:** 2025-10-14

**Authors:** Muharrem Uçar, Mukadder Şanlı, Sezai Aktürk, İlham Gülçek, Feray Akgül Erdil

**Affiliations:** 1 İnönü University Faculty of Medicine, Department of Anaesthesiology and Reanimation, Malatya, Türkiye; 2 İnönü University Faculty of Medicine, Department of Thoracic Surgery, Malatya, Türkiye

**Keywords:** Complement C1 inhibitor protein, fresh frozen plasma, hereditary angioedema, tracheal stenoses

## Abstract

Hereditary angioedema (HAE) causes recurrent angioedema attacks in the oropharynx, larynx, face, and other regions due to bradykinin overproduction as a result of C1 esterase inhibitor deficiency. Surgical interventions requiring general anaesthesia might trigger HAE attacks. Laryngeal angioedema is the most important cause of perioperative mortality. Tracheal dilatation was performed by rigid bronchoscopy in our patient with type 1 HAE, because of tracheal stenosis due to prolonged intubation, which occurred after the attack. The patient was administered 2x500 IU C1 esterase inhibitor approximately 24 hours before rigid bronchoscopy. No complication developed after the first procedure. Two months later, tracheal dilatation was repeated and 2x500 IU C1 esterase inhibitor was administered. While the patient was followed up in the intensive care unit, significant oedema developed in the facial area, especially the tongue and lips, approximately 10 hours after the procedure. Our patient also had stridor due to airway obstruction. The patient was treated with 1000 IU C1 esterase inhibitor and 3 units of fresh frozen plasma (FFP). After FFP, edema started to regress. The patient was discharged after symptoms improved. The patient should be monitored in the intensive care unit for a minimum of 48 hours to monitor for postoperative laryngeal oedema.

**Main Points**

• Hereditary angioedema causes recurrent angioedema attacks in the oropharynx, larynx, and face.

• Laryngeal angioedema is the most important cause of perioperative mortality.

• Hereditary angioedema may develop even after uneventful anaesthesia and surgery.

• Hereditary angioedema requires good preoperative preparation, careful intraoperative follow-up, and postoperative care.

## Introduction

Hereditary angioedema (HAE) is a rare autosomal dominant disease characterized by C1 esterase inhibitor deficiency, leading to increased bradykinin production. C1 esterase inhibitor deficiency causes recurrent angioedema attacks in the oropharynx, larynx, face, and other areas via bradykinin. This angioedema can be seen in the larynx during upper airway manipulation such as tracheal intubation. Laryngeal angioedema is the most important cause of perioperative mortality. Its treatments are either the C1 esterase inhibitor or fresh frozen plasma (FFP).^1, 2^

In this case report, we aimed to present the anaesthesia management of a patient with HAE, for whom tracheal dilatation was planned due to tracheal stenosis.

## Case Report

The patient was a 43-year-old female with a weight of 105 kg and a height of 165 cm. The thoracic surgeon planned rigid bronchoscopy under general anaesthesia with the preliminary diagnosis of tracheal stenosis. Our patient was diagnosed with type 1 HAE. In the anamnesis of the case, she had two attacks due to HAE. In the first case, improvement was achieved with medical treatment. After the second attack, the patient was intubated due to spontaneous swelling of the tongue, and then a tracheostomy was opened. She remained intubated for 18 days. Her physical examination revealed no symptoms other than shortness of breath. Laboratory findings were as follows. Glucose: 135 mg dL^-1^, Hgb: 12.6 g dL^-1^, Sed: 25, and other parameters were normal. Before rigid bronchoscopy, 2x500 IU C1 esterase inhibitor (CINRYZE 500 IU/5 mL IV) was administered to the patient approximately 24 hours before the procedure. The patient was standard monitored in the operating room and, considering that difficult intubation may develop due to laryngeal edema, it was decided that a C-MAC D blade video laryngoscope, tracheostomy preparation, C1 esterase inhibitor, and FFP were to be kept ready in the room. A 20 G vascular access was established for the patient. 1 mg kg^-1^ lidocaine, 1 µg kg^-1^ fentanyl, 2 mg kg^-1^ propofol, and 0.6 mg kg^-1^ rocuronium were administered IV through the vascular access. The patient was ventilated using a face mask to avoid creating pressure on the face. After muscle relaxation, the thoracic surgeon used a rigid bronchoscope no. 6.5. The vocal cords were traversed and the trachea was entered. The standard anaesthesia circuit was connected to the side inlet of the rigid bronchoscope, and ventilation was provided with 100% oxygen with intermittent positive pressure. Anaesthesia was maintained with intermittent IV pushes of propofol and rocuronium. During the procedure, the patient’s mean arterial pressure was between 60-70 mmHg and SpO_2_ was between 95-98%. A stenosis of approximately 2 cm in length, 1 cm past the vocal cords, was observed, and dilation was performed with a rigid bronchoscope number no 7.5. After the procedure, the patient was awakened without any problems after applying 2 mg kg^-1^ sugammadex, and was sent to the intensive care unit. The patient’s general condition was good during follow-up and she was discharged. Two months later, rigid bronchoscopy was successfully performed again to dilate the trachea. The same prophylactic treatment of 2x500 IU C1 esterase inhibitor was applied before the operation. Approximately 10 hours after the operation, the patient in the intensive care unit developed significant edema in the facial area, primarily the tongue and lips. In addition, our patient had stridor due to airway stenosis. The patient was treated with 1000 IU C1 esterase inhibitor and 3 units of FFP. After FFP, edema started to regress. The patient was discharged after her symptoms improved.

## Discussion

Operations requiring general anaesthesia might trigger HAE attacks. The use of inhalation and intravenous anaesthetic drugs in cases with bradykinin-mediated angioedema is not contraindicated.^3^ We used propofol as an anaesthetic agent in the induction and maintenance of our patient and did not encounter any problems.

Upper airway manipulation and tracheal intubation may trigger upper airway and laryngeal angioedema. Laryngeal edema is the most serious complication of HAE.^3, 4^ It was reported that life-threatening airway angioedema attacks are rare, although they may occur in patients receiving short-term prophylaxis and in those who have previously received anaesthesia without problems.^3^ We did not encounter any problems in anaesthesia management.

Treatment of HAE: the aim of the treatment includes increasing C1-INH levels and inhibiting the effects of kallikrein, bradykinin, and plasmin.^3, 5, 6^ If concentrate is not available, it is recommended to use plasma treated with solvent/detergent 1-6 hours before the procedure, or if this is not available, it is recommended to use treatment with FFP.^2^ One unit of C1-INH concentrate corresponds to the average amount of C1-INH found in 1 mL of fresh normal plasma.^6^

Trigger sensitivity varies among patients with HAE. While triggers vary from patient to patient, common triggers include trauma, medical procedures, and stress. Physical trauma is a prominent and common threat to the larynx.^4^ It has been reported that laryngeal oedema may occur more than 2 days later, and therefore postoperative monitoring is important.^2, 7, 8, 9^ Since FFP contains all of the clotting factors and C1 INH, it can be used in the treatment of acute HAE attacks.^4, 10, 11^ FFP is used to treat acute HAE attacks, especially in countries where C1-INH concentrate is not available.^9, 11^ The recommended dosage for FFP application in the treatment of HAE attacks is 10-20 mL kg^-1^, with an average use of 1-2 units.^7, 10, 11^ Our patient also developed acute and severe angioedema in the intensive care unit approximately 10 hours postoperatively. The treatment of acute HAE, we administered the second dose of C1 INH and FFP to our patient. Fresh frozen plasma carries the risk of worsening attacks with the addition of contact pathway proteins.^1, 3, 6, 10, 11^ The FFP we applied to our patient did not worsen the attacks, but instead treated them, and we did not encounter any side effects.

## Conclusion

HAE requires caution because of the risk of developing laryngeal edema. It should be anticipated that postoperative HAE may develop even after an uneventful anaesthetic event and surgery. It should be kept in mind that these cases require thorough preoperative preparation, careful intraoperative follow-up, and postoperative care.

## Ethics

**Informed Consent:** The complete procedure was explained to the patient, and informed consent was obtained.
